# Gold Nanoparticles Supported on Urchin-Like CuO: Synthesis, Characterization, and Their Catalytic Performance for CO Oxidation

**DOI:** 10.3390/nano10010067

**Published:** 2019-12-27

**Authors:** Feng Dong, Yuan Guo, Dongyang Zhang, Baolin Zhu, Weiping Huang, Shoumin Zhang

**Affiliations:** Department of Chemistry, Key Laboratory of Advanced Energy Material Chemistry (MOE), TKL of Metal and Molecule Based Material Chemistry, Nankai University, Tianjin 300071, China; dongfeng12839001@163.com (F.D.); 2120190685@mail.nankai.edu.cn (Y.G.); 2120180940@mail.nankai.edu.cn (D.Z.); hwp914@nankai.edu.cn (W.H.)

**Keywords:** urchin-like CuO microsphere, Au-loaded catalyst, catalytic activity, CO oxidation

## Abstract

Gold catalysts have been studied in-depth due to their unique activities for catalytic CO oxidation. Supports have intrinsic motivation for the high activity of gold catalysts. Thermally stable urchin-like CuO microspheres, which are potential support for gold catalysts, were prepared by facile solution-method. Then gold nanoparticles were loaded on them by deposition-precipitation method. The obtained gold catalysts were characterized by SEM, XRD, TEM, BET, ICP, and XPS. Their catalytic activity for CO oxidation was also evaluated. TEM results revealed that the gold nanoparticles with small sizes were highly distributed on the CuO surface in Au_1.0_/CuO-300. XPS observations demonstrated that the gold species in Au_1.0_/CuO-300 was of metallic state. Among the as-prepared catalysts, the Au_1.0_/CuO-300 catalyst displayed the best performance for CO oxidation and achieved 100% CO oxidation at 80 °C. It kept 100% conversion for 20 h at a reaction temperature of 180 °C, and showed good reusability after three reaction-cycles. The possible catalytic mechanism of Au_1.0_/CuO-300 catalyst for CO oxidation was also briefly proposed.

## 1. Introduction

Carbon monoxide (CO) is not only one of the major environment pollutants, but also is one serious threat to human health [1]. It is mainly generated by the incomplete combustion of fossil fuels involved in industrial process and living heating. Thus, it is necessary to timely eliminate CO in the air by developing highly active catalysts, which becomes one considerable research field due to its significant applications in industrial gas treatment, automotive exhaust purification, fuel cells, and so on [2]. It has been demonstrated that noble metals (such as Au) exhibit excellent activities for catalytic CO oxidation. Especially, the nanoscale noble-metals on metal-oxide semiconductor can efficiently obtain enhanced catalytic activity, which might be assigned to synergetic effect between support and gold particles. This effect could stimulate the charge delivery and energy transfer [3].

In the late 1980s, it is confirmed that Au/metal-oxide catalyst possessed high catalytic performance in the low temperature for CO oxidation [4]. The main studies of Haruta are classic researches over CO oxidation [5,6]. Haruta reported that Au/M_X_O_Y_ (M = Fe, Co, Ni) catalysts possessed high activity for CO oxidation even at the low temperature of −70 °C [6]. Moreover, gold catalyst also has been applied to other reactions extensively [7]. It has been demonstrated that gold particle with 2–5 nm size is most active [4]. However, it is generally accepted that the catalytic activity of gold catalyst is also strongly affected by preparation method [8], gold-support interaction [9], pretreatment conditions [10], reaction atmosphere [11], and especially the selection of the support [10].

On the other hand, the performance of gold catalyst also relates to the capability of Au nanoparticles to adsorb CO molecules [12]. In addition, it is now well acknowledged that the metal oxides support plays a significant role in the adsorption and activation of oxygen molecules during catalytic CO oxidation [13]. Because of the strong influence of support on the activity of gold catalyst, metal oxides supports were classified into active (CuO, MnO_2_, Fe_2_O_3_, TiO_2_, CeO_2_) and inert supports (Al_2_O_3_, La_2_O_3_, SiO_2_, MgO) in the light of their reducibility [14,15]. The gold catalysts with active supports often exhibit excellent activity for CO oxidation.

Especially, CuO support has attracted intensive attention for CO oxidation due to its good catalytic activity and abundant resources [16,17,18]. In the early stage, Hutchings and co-workers prepared Au/CuO catalysts by coprecipitation (CP) method, which has highly sustained activity during CO oxidation process [19]. Up to now, it has been well-proven that the size and morphology of CuO support could greatly affect the performance of catalyst. Through various methods, various CuO microstructures have been prepared, such as nanoflowers [20], nanoribbons [21], nanorods [22], and nanosheets [23]. Among these preparations of CuO supports (including hydrothermal synthesis [24], thermal oxidation [25], ultrasound-assisted method [26], solution-phase method [27], and microwave-assisted method [28]), solution-phase method is a kind of simple and low-energy consumptive preparation one. Moreover, the adjusting of preparation conditions (such as temperature, fabrication time, precursors, solution concentration, and pH) also plays an important role in controlling the CuO morphology [26,29,30].

As have been reported, the catalytic capability of Au/CuO catalysts is affected by the morphology of CuO support [16,17,18,31]. For instance, Lei et al. [16] prepared Au/CuO nanosheets, which exhibited good performance for CO oxidation at low temperature. Zhang [17] used a porous CuO ribbons modified by gold nanoparticles as CO oxidation catalyst. The CO full-conversion on this catalyst occurred at 180 °C.

In particular, urchin-like CuO architectures, assembled with one- or two-dimensional nanostructures, have excellent properties for various applications (such as gas sensors, battery, photocatalysts, etc.) due to their strong supplying capacity of active sites, high thermal stability and advanced capability of electron transfer [32,33]. Although the urchin-like CuO support could greatly promote the catalytic properties of noble metal, the researches over the urchin-like CuO support combined with the noble metals has been rarely studied. Ying et al. [34] reported urchin-like Pd@CuO–Pd electrocatalysts displayed high electrocatalytic performance and selectivity for glucose oxidation. Yan et al. [35] suggested that the gold could enhance the sensing performance with low detection limit over urchin-like ZnO-Au@CdS microspheres. If the active gold species supported on the urchin-like metal oxides, one composite with superior property can be obtained. However, so far there are few studies over Au nanoparticles supported on the urchin-like CuO microspheres for catalytic CO oxidation.

Herein, urchin-like CuO, microspheres were prepared under a controlled approach at room temperature. The well-defined urchin-like CuO microspheres were obtained by the optimization of NaOH drop-time and the possible formation mechanism was also discussed. Furthermore, a series of Au_X_/CuO (x = 0.5, 1.0, 1.5 wt %) catalysts were prepared by deposition-precipitation method. The samples were characterized by BET, SEM, XRD, TEM, ICP, and XPS. Their catalytic activity for CO oxidation was further investigated. The activity results showed that Au_1.0_/CuO-300 catalyst had the best catalytic performance and best high-temperature stability among the as-prepared catalysts. The possible reason for enhanced activity of Au_1.0_/CuO-300 should be the synergistic interaction between gold species and CuO. The catalytic mechanism of Au_1.0_/CuO-300 catalyst for CO oxidation were reasonably proposed.

## 2. Materials and Methods

All chemicals were of analytic grade and used without pretreatment. Deionized water was used throughout the experiments.

### 2.1. CuO Support Preparation

The CuO supports were prepared by surfactant-assisted solution method. Typically, an ethanol solution of TOAB (Tetraoctylammonium bromide, 40 mL, 0.05 M) was added to aqueous solution of Cu(NO_3_)_2_·3H_2_O (20 mL, 0.05 M) under constant stirring for 5 min at room temperature (solution A). Afterwards, NH_3_·H_2_O (25–28%, 5 mL) was fast injected into solution A (solution B). Then aqueous NaOH solution (10 mL, 1.00 M) was added drop-wise into the solution B with different time of dropping (5 min, 15 min, 25 min, 35 min, 45 min, and 55 min) under vigorous stirring, and then the mixture was kept constant stirring for 1.5 h at room temperature. The produced blue precipitates were filtered, centrifuged, washed, with distilled water and ethanol for several times before drying at 60 °C in vacuum. Finally, the samples were calcined in air at 500 °C for 1 h. The prepared samples were denoted as CuO-X, in which X represented the drop-wise time of NaOH solution.

### 2.2. Catalyst Preparation

Deposition-precipitation method was carried out to prepare Au_X_/CuO catalysts, in which x represented the theoretical Au loading on CuO (x = 0.5, 1.0 and 1.5 wt %). 0.4 g CuO-45 supports were dispersed in 100 mL deionized water and the suspension was kept stirred for 5 min. Then different amounts of HAuCl_4_ solution (0.01 M) was added to the above suspension respectively. The suspension was adjusted to pH = 7 with NH_3_·H_2_O (25–28%), and then stirred for 12 h at room temperature. Afterwards, the suspension was centrifuged and washed with deionized water to remove NH_4_Cl. After dried at 80 °C overnight (denoted as Au_X_/CuO-80), all the products were calcined in air at 300 °C for 2 h (denoted as Au_X_/CuO-300). Au_1.0_/CuO-80 samples were calcined in air at 400 °C or 500 °C for 2 h, respectively (denoted as Au_1.0_/CuO-400 or Au_1.0_/CuO-500).

### 2.3. Characterization

The morphologies of samples were observed using ZEISS MERLIN compact (Field Emission) X-650 scanning electron microscope (SEM) (Zeiss, Jena, Germany) operating at 25 kV. The X-ray diffraction (XRD) experiments were carried out using a Rigaku D/Max-2500 X-ray diffractometer (CuKαλ = 0.154 nm) to investigate the crystal phase. Transmission electron microscopy (TEM) images of the samples were obtained using a JEM-2100 transmission electron microscopy working at 200 kV. The chemical composition and oxidation state of elements on the surface of samples were identified by X-ray photoelectron spectroscopy (XPS) (Ulvac-Phi, Chigasaki, Japan) using an Al X-ray source (Al Ka150 W, PHI 5000 Versa Probe), and the binding energy was calibrated by taking C 1s peak at 284.6 eV as reference. Brunauer–Emmett–Teller (BET) specific surface area of samples were measured by nitrogen adsorption at liquid N_2_ temperature on Micromeritics Tristar II 3020 apparatus made in Germany.

The actual gold loadings in samples were identified by inductively coupled plasma (ICP) on an IRIS Advantage instrument. The actual gold loadings of Au_0.5_/CuO-300, Au_1.0_/CuO-300, and Au_1.5_/CuO-300 were 0.36, 0.85, and 1.39 wt %, respectively. These results revealed that the actual gold loadings in all catalysts were slightly lower than the nominal ones with the loading efficiencies in the range of 72–93%. Although gold was lost during the preparation process, the gold was easily supported on the CuO surface.

### 2.4. Catalytic Activity

Catalytic activities of catalysts for CO oxidation were evaluated under atmospheric pressure in a fixed-bed flow reactor. A stainless steel tube with an inner diameter of 8 mm was used as the reactor and 200 mg sample catalyst was diluted by chemically inert quartz sand (17.6 g). Reaction gas containing of 10% CO and balanced air was flowed through reactor at a total flow rate of 36.3 mL/min. The catalytic activity of sample was recorded at a heating ramping rate of 5 °C/min and kept at the reaction temperature for 17 min. Then effluent gases were analyzed on-line on a GC-508A gas chromatography using H_2_ as carrier gas. To test the catalysts’ activities, the conversion of CO was defined by

(1) $$\mathrm{CO}\;\mathrm{Conversion}=\frac{\left[{\mathrm{CO}}_{2}\right]}{\left[\mathrm{CO}\right]+\left[{\mathrm{CO}}_{2}\right]}\times 100\%$$

## 3. Results

### 3.1. Effect of Time of Dropping of NaOH on CuO Support

**Figure 1:**
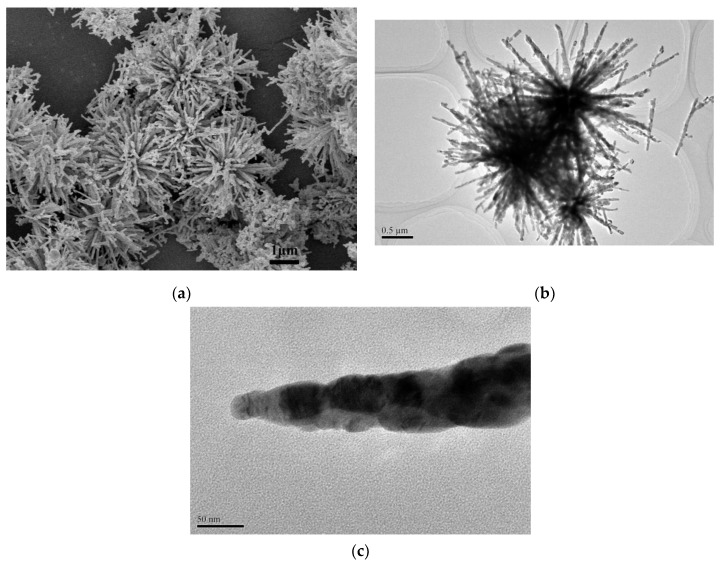
SEM image (**a**) and TEM images (**b**,**c**) of CuO-45 supports.

In order to obtain more structural details about the morphology of as-synthesized CuO-45 supports, their SEM and TEM images were shown in Figure 1. It can be observed that the well-defined urchin-like CuO formed with a diameter of about 4 μm and rough surface. It could be further seen that the urchin-like CuO was assembled by many nanorods with thickness of around 60 nm and 1–1.5 μm in length. Interestingly, Figure 1c displays that the oriented aggregation growth of nanoparticles-by-nanoparticles led to the formation of CuO nanorods. Although the diameters of nanorods varied from the bottom to the top, the bottom of rods are connected together and rooted in the center of urchin-like CuO, exhibiting high regularity and uniformity. Moreover, after the samples were calcined at 500 °C (Figure 1b), the urchin-like structure was still well maintained due to its high thermal stability.

**Figure 2:**
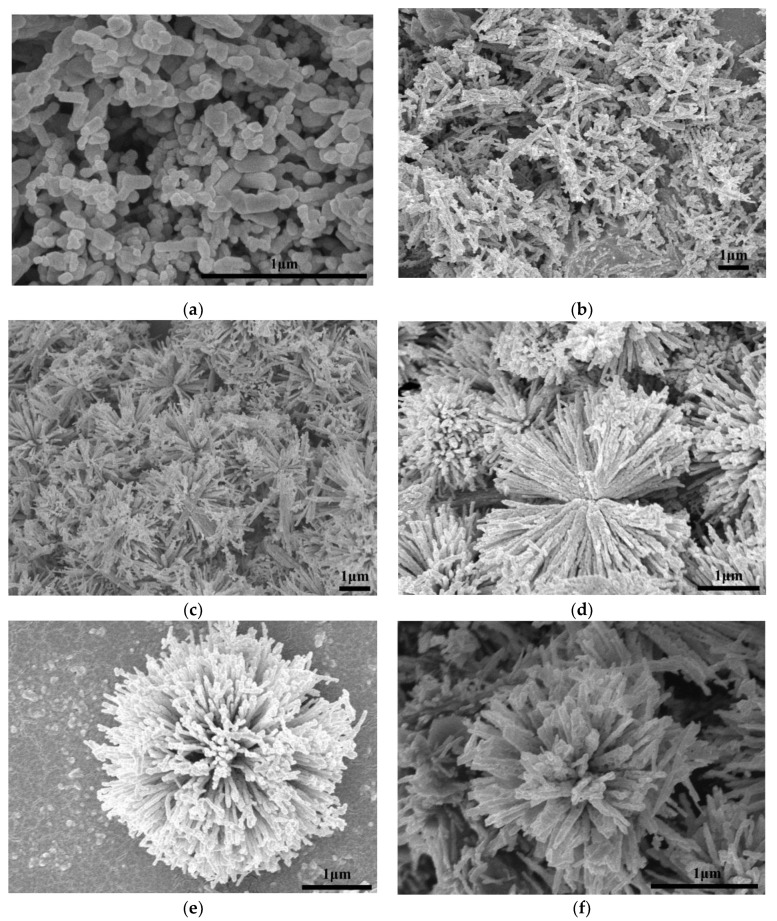
Morphology of CuO microspheres obtained at room temperature with varied time of dropping of NaOH: 5 min (**a**), 15 min (**b**), 25 min (**c**), 35 min (**d**), 45 min (**e**), and 55 min (**f**).

In order to investigate the crystal growth process of urchin-like CuO microspheres, the time of dropping of NaOH solution dependent experiments were performed at room temperature. Figure 2 shows the effect of time of dropping of NaOH on the morphology evolution of CuO microspheres.

When the time of dropping of NaOH was 5 min (Figure 2a), a large number of irregular nanoparticles with diameter of around 45–200 nm appeared. When the time of dropping of NaOH was 15 min (Figure 2b), the nanoparticles disappeared and major products were highly agglomerated nanorods. Further extending the NaOH drop-time time to 25 min (Figure 2c), the nanorod-bundle structures formed on a large scale, which were composed of several nanorods. When the time of dropping of NaOH was 35 min (Figure 2d), the formed double trumpet-like patterns were consisted of orderly stacked nanorods. Also, these rods grew from the same center point. When the time of dropping of NaOH was extended to 45 min (Figure 2e), the urchin-like CuO with intense uniformity and regularity was produced finally. These microspheres were actually all assembled by the nanorods. If the time of dropping of NaOH was 55 min (Figure 2f), the slightly agglomerated urchin-like CuO microsphere were formed, while comparatively having asymmetrical size and irregular shape because of the dissolution/crystallization rule [36].

Above experimental results illustrate that the time of dropping of NaOH could significantly affect the morphology, which might be due to the fact that the time of dropping of NaOH affected the nucleation and growth processes of nanocrystals. Hence, the time of dropping of NaOH plays a key role in controlling the urchin-like morphology of CuO microsphere, and the time of dropping of NaOH (45 min) is the optimal.

### 3.2. Formation Mechanism of Urchin-Like CuO

**Scheme 1:**
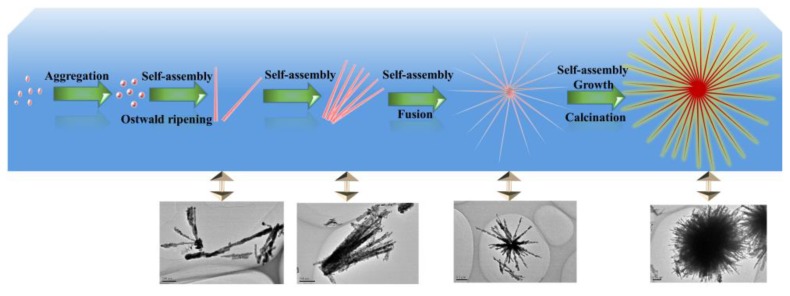
Schematic illustration of the possible formation mechanism for the urchin-like CuO structure.

From the above results and reported references [30], the possible formation process of urchin-like CuO architectures could be schematically divided into the following steps (Scheme 1):

(i) Formation of the primary nanocrystals: In initial reaction time, the Cu(OH)_2_ nucleation was formed by the equilibria of complex ions in solution. In addition, the chemical process for formation of CuO microsphere is ascribed in Equations (1)–(5) as follows
  Cu^2+^ + 6H_2_O ⇋ [Cu(H_2_O)_6_]^2+^ (2)
  [Cu(H_2_O)_6_]^2+^ + 4NH_3_ ⇋ [Cu(NH_3_)_4_]^2+^ + 6H_2_O (3)
  [Cu(NH_3_)_4_]^2+^ + 4OH^−^ ⇋ [Cu(OH)_4_]^2−^ + 4NH_3_ (4)
  [Cu(OH)_4_]^2−^ → Cu(OH)_2_↓ + 2OH^−^ (5)
  Cu(OH)_2_ → CuO + H_2_O (6)

In the first step, upon the vigorous stirring, TOAB molecules were homogeneously dispersed in the ethanol solution. Then, Cu(NO_3_)_2_·3H_2_O and NH_3_·H_2_O were successively added to the above aqueous solution, forming the [Cu(H_2_O)_6_]^2+^ ions due to the six-coordinated structure of Cu^2+^ ion, according to Equation (1). Because the NH_3_ is a stronger ligand than H_2_O, the four NH_3_ molecules linked to Cu^2+^ plane and other two NH_3_ molecules existed in its axis to form Cu(NH_3_)_6_^2+^ structure by replacing H_2_O molecules. However, the binding energies of two NH_3_ existed in axis were lower than those of NH_3_ linked to plane, implying that the Cu(NH_3_)_4_^2+^ square units were actually formed in the aqueous solution [37,38] and can be seen from Equation (2). Especially, the [Cu(NH_3_)_4_]^2+^ as activation catalyst in the solution not only might promote crystal formation and lead to a specific orientation [37], but also is a key factor for the different assembling patterns of Cu(OH)_2_ by varying the reaction kinetics [30,37,38,39].

Subsequently, upon slowly adding NaOH during 45 min, OH^−^ ions were gradually attracted to Cu^2+^ ions by replacing NH_3_ in the [Cu(NH_3_)_4_]^2+^ complex, which resulted in the formation of square-planar [Cu(OH)_4_]^2−^ units according to Equation (3). This ionic attraction could accelerate a higher growth rate via Cu-OH bonding, which contributes to the formation of oriented structures. Then, [Cu(OH)_4_]^2−^ units had a strong tendency to form Cu(OH)_2_ units according to Equation (4) [40], which also absorbed the TOA^+^ headgroup of TOAB molecules. If NaOH was added to the solution too quickly, the [Cu(OH)_4_]^2−^ units would convert into the aggregated CuO particles directly through high temperature, which would change the kinetic growth of nanostructure by spoiling reaction equilibrium. When the Cu(OH)_2_ units gradually grew up to the nuclei size, these nucleus attracted more [Cu(OH)_4_]^2−^ units by the TOA^+^ head-group of TOAB and further fused into polycrystalline cores. Finally, small Cu(OH)_2_ particles were obtained for energy minimization in liquids [41]. Hence, Equation (3) is a key reaction, which can efficiently affect the nucleation and growth of nanocrystals through precisely controlling the supply-speed of OH^−^ ions [30,42].

(ii) Growth of the secondary structure: In highly alkaline conditions, it was easy to form the pristine nanorod via the self-assembly of Cu(OH)_2_ nanoparticles [37] and Ostwald ripening effect [43,44]. This should be attributed to the Van der Waals forces for minimizing the overall surface free energy [45]. In addition, TOAB also could facilitate the formation of nanorods via oriented attachment.
(iii) Formation of the three dimensional structure: With the assistance of TOAB, the nanorods self-assembled into urchin-like structures through the Van der Waals force and driving force of hydrogen bond between the OH^−^ groups of Cu(OH)_2_ [37,46], followed by the growth and crystallographic fusion into bigger urchin-like structures for the decrease of Gibbs free energies of whole system [38,47]. Finally, the reaction system was inclined to be the thermodynamically stable state. During the process of the nanostructure formation, TOAB molecules, acted as a growth-directing agent, could modulate spontaneous self-assembling from nanocrystals to urchin-like structures.

In short, it could be concluded that the whole transformation process consisted with the thermodynamically driven spontaneous process, which was from Cu(OH)_2_ nanoparticles to Cu(OH)_2_ nanorods and then to assemble urchin-like Cu(OH)_2_. The well-crystallized urchin-like CuO microsphere was finally formed through calcination (see Equation (5)).

### 3.3. XRD

**Figure 3:**
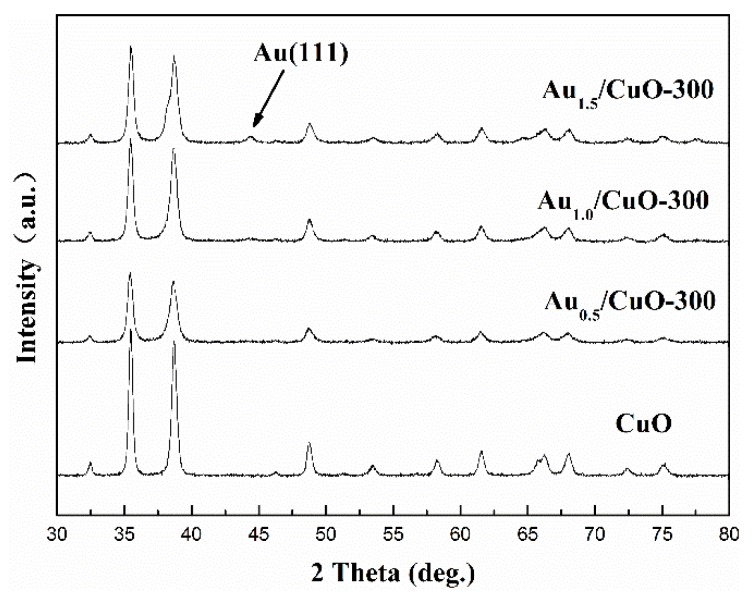
Full XRD patterns of CuO support and Au_X_/CuO-300 catalysts with various gold loadings.

Figure 3 shows that the XRD patterns of CuO support and Au_X_/CuO-300 catalysts (Au content: 0.5, 1.0, and 1.5 wt %). The peaks (35.42°, 35.54°, 38.71°, 38.90°, 48.72°, and 61.52°) of CuO support with features of high-intensity corresponding to (0 0 2), (1 1-1), (1 1 1), (2 0 0), (2 0-2), and (1 1-3) crystal planes of CuO (JCPDS 48-1548), indicating the calcined CuO support were well-crystalline [48]. No other peaks of impurities in the XRD spectra of CuO appeared, implying that the CuO had been completely produced. For Au_1.5_/CuO-300 sample, the indexed peaks of 38.2°, which overlaps with peak of CuO-(111) plane, and 44.5° corresponding to (1 1 1) and (2 0 0) planes of Au^0^ (JCPDS 04-0784) respectively can be observed. It could prove that Au_1.5_/CuO-300 sample actually contained gold species. However, no visible peaks of gold can be observed in the Au_0.5_/CuO-300 sample and Au_1.0_/CuO-300 sample, which probably due to the relatively low contents of gold and highly dispersed small gold nanoparticles. In addition, the XRD peaks of Au_X_/CuO-300 samples are broadening compared to that of CuO sample, which suggested that the gold species reduced the crystallinity of samples.

### 3.4. TEM

**Figure 4:**
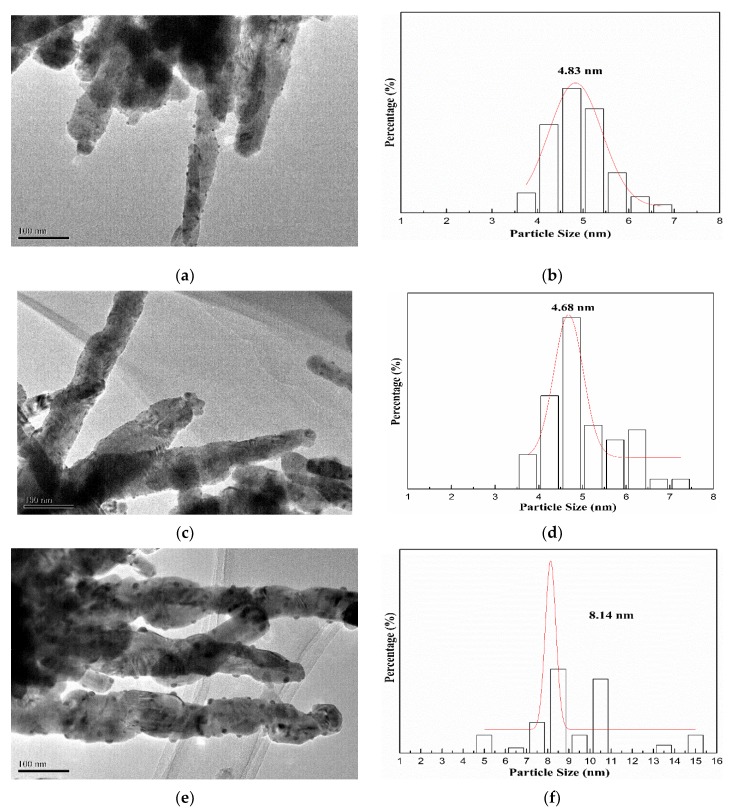
Size distribution histograms and TEM images of Au_0.5_/CuO-300 (**a**,**b**), Au_1.0_/CuO-300 (**c**,**d**), Au_1.5_/CuO-300 (**e**,**f**).

**Figure 5:**
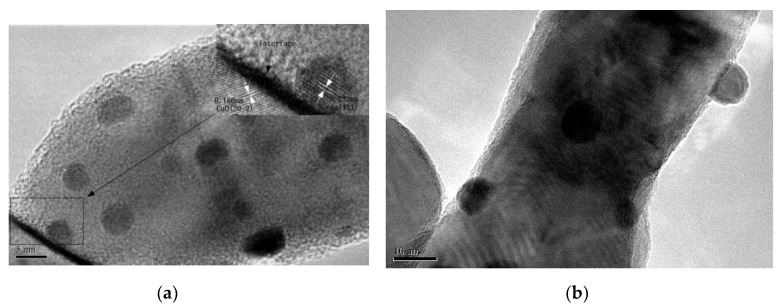
HR-TEM images of Au_1.0_/CuO-300 (**a**) and Au_1.0_/CuO-500 (**b**).

The morphology and particle size distribution of Au_X_/CuO-300 catalysts were shown as Figure 4a–f by TEM analysis. Compared with other samples (Figure 4a,e), the gold nanoparticles with the mean size (4.68) nm for Au_1.0_/CuO-300 sample were homogeneously and highly distributed on the CuO surface. Accordingly, the mean gold nanoparticle size of Au_1.0_/CuO-300 sample was also the smallest among all the samples. These results might be assigned to the low gold loading. Besides, the mean Au nanoparticle size of 8.14 nm for Au_1.5_/CuO-300 was the largest among all the samples, indicating the increase of Au loading resulted in the increase of gold particle size. To further explore the detailed nanostructure of Au_1.0_/CuO-300 sample, HR-TEM image of Au_1.0_/CuO-300 was obtained (Figure 5a). The measured interplanar distance of 0.232 and 0.184 nm corresponded to the (111) plane of Au and (202-2) plane of CuO, respectively. The clear interface between Au and CuO is also evident in the image. As shown in Figure 5b, the gold nanoparticles of Au_1.0_/CuO-500 sample obviously grew to around 10 nm after the 500 °C calcination treatment, which should be attributed to the aggregation of gold nanoparticles induced by the calcination process at high temperature. That possibly leads to the decrease of catalytic activity of the supported gold catalyst.

### 3.5. XPS

**Figure 6:**
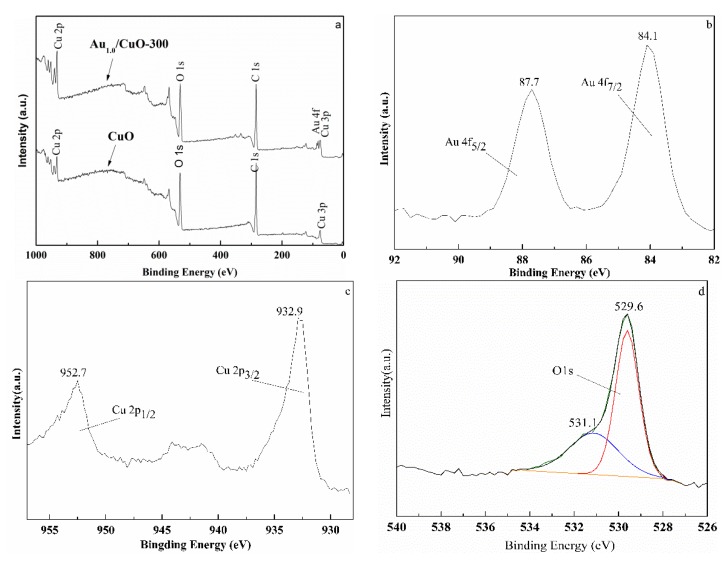
Wide XPS spectrum of CuO and Au_1.0_/CuO-300 (**a**) and high-resolution XPS spectra of Au_1.0_/CuO-300: Au 4f peaks (**b**), Cu 2p peaks (**c**), and O 1s peaks (**d**).

In order to explore the surface components and chemical states of elements in samples, XPS analysis results were displayed in Figure 6. Compared with the XPS survey spectra of CuO, the spectra of Au_1.0_/CuO-300 have new signals of Au 4f (Figure 6a), indicating the Au_1.0_/CuO-300 sample contains gold element. The signal of carbon in both samples might due to the used TOAB during the preparation or XPS measurement itself.

Figure 6b–d display the high-resolution XPS spectra of Au, O and Cu of Au_1.0_/CuO-300 sample. The Au 4f_7/2_ peak at around 84.1 eV and Au 4f_5/2_ peak at 87.7 eV (Figure 6b) are both typical values for the metallic state Au [49]. No peaks corresponded to oxidized gold species were detected at 85.5 and 86.3 eV, implying all the Au^3+^ ions were completely reduced to the Au^0^. Obviously, the Au 4f_7/2_ peak had the 0.1 eV positive shift of BE compared to the Au 4f_7/2_ BE (84.0 eV) of metallic Au^0^. This phenomenon might result from the smaller gold nanoparticle size [50,51,52] and certain synergistic interaction between the Au and CuO [53].

Double peaks at 932.9 eV for Cu 2p_3/2_ and 952.7 eV for Cu 2p_1/2_ along with shakeup satellite peak at 942.5 eV indicate the presence of Cu^2+^ in the CuO support (Figure 6c). The O 1s region peak at around 529.6 eV can be attributed to O^2−^ (Figure 6d), which belong to the highly polarized oxide ions on the CuO surface [54]. Moreover, the weak and broad shoulder peak at 531.1 eV (Figure 6d) was likely attributed to adsorbed oxygen or hydroxyl in Au_1.0_/CuO-300 sample [54,55].

### 3.6. BET

**Figure 7:**
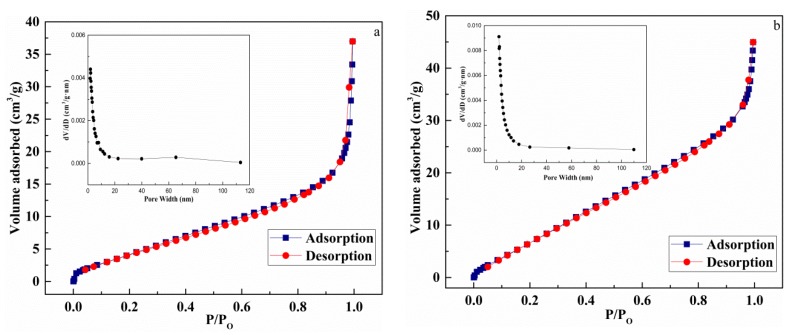
N_2_ adsorption–desorption isotherms and pore size distributions of CuO (**a**) and Au_1.0_/CuO-300 (**b**).

Figure 7 presents the N_2_ adsorption–desorption isotherms and pore size distributions of CuO (a) and Au_1.0_/CuO-300 (b). The isotherms of CuO and Au_1.0_/CuO-300 was the same type. The BET surface area of Au_1.0_/CuO-300 and CuO was 35.3 and 18.0 respectively.

### 3.7. Catalytic Activity

#### 3.7.1. Effect of Au Contents

**Figure 8:**
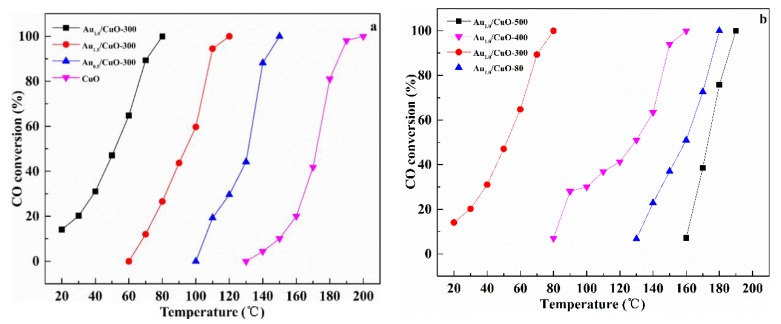
Catalytic activities of CuO and Au_X_/CuO-300 with different Au loadings (**a**); Au_1.0_/CuO calcined at different temperatures (**b**).

CO oxidation was used as a probe reaction to study catalytic properties of the prepared catalysts and the conversion of CO oxidation was monitored as a function of reaction temperature. Figure 8a shows the effect of Au content on the activities of catalysts. It is worth pointing out that 10% CO was used in this work while 1% CO was used in most studies.

The CuO had low activity from 140 °C to 200 °C. After gold nanoparticles were supported on the CuO, the temperature for full CO conversion was obviously decreased. Therefore, it could be believed that gold nanoparticles supporting the urchin-like CuO is a rational way to significantly enhance the catalytic activity of CuO. Catalytic activities of samples were estimated by T_100%_ (T_100%_: the temperature at which 100% CO conversion was obtained) in the order: Au_1.0_/CuO-300 > Au_1.5_/CuO-300 > Au_0.5_/CuO-300 > CuO. Obviously, the Au_1.0_/CuO-300 had the best catalytic activity among all the as-prepared catalysts, which was activated at about 20 °C and rapidly increased to 100% CO conversion at 80 °C. To compare the catalytic performance more precisely, the calculated specific rate of Au_1.0_/CuO-300 was about 1.15 mol_CO_ g_Au_^−1^ h^−1^, which was 2.4 times than 0.48 mol_CO_ g_Au_^−1^ h^−1^ of Au/TiO_2_ catalyst (World Gold Council) at the same reaction temperature. This was likely result from the high dispersion of Au nanoparticles on the CuO (See TEM results) and synergistic interaction between gold and CuO. This result was consistent with previous study that the loading amount of gold on the CuO could affect the catalytic activity of catalyst toward CO oxidation. The activity of the gold catalyst not only depends on the size of the gold particles, but also depend on the gold content. Compared with Au_1.0_/CuO-300, Au_0.5_/CuO-300 had fewer active sites due to its low Au content, which lead to its lower activity. Additionally, excessive Au species was easily to be aggregated into the larger particles and further blocked the active sites, which resulted in the relatively low activity of Au_1.5_/CuO-300 [56]. As a result, the optimal Au loading of Au_X_/CuO-300 catalyst is 1.0 wt %.

#### 3.7.2. Effect of Calcination Temperatures

Figure 8b shows the effect of calcination temperature on the catalytic activities of catalysts. The results displayed a sharp increase in the activity of catalyst with the raising of calcination temperature (from 80 °C to 300 °C). However, when calcination temperature was increased from 300 °C to 500 °C, the T_100%_ of catalyst was obviously increased from 80 °C to 190 °C. The T_100%_ of Au_1.0_/CuO-300 was 80 °C, while T_100%_ of Au_1.0_/CuO-500 and T_100%_ of Au_1.0_/CuO-80 were 190 °C and 180 °C respectively. Therefore, the Au_1.0_/CuO-300 was more active than other catalysts for CO oxidation. This result also means that the calcination at 500 °C seriously deactivated the CuO-supported gold catalyst because of the enlarged Au particles, as observed in Figure 5b, resulting in the decrease of active sites for CO oxidation. As for the previous studies, gold nanoparticle size along with the metal–support interaction play important role in determining the activity of catalyst. Therefore, it could be reasonably deduced that gold particle size in Au_1.0_/CuO-300 was relatively small and the gold-support interaction is relatively strong, leading to the high activity of Au_1.0_/CuO-300. Briefly, the calcination treatment had a significant influence on the catalytic activity of CuO-supported gold catalysts and the optimal calcination temperature was 300 °C.

#### 3.7.3. Reusability and Stability Test

**Figure 9:**
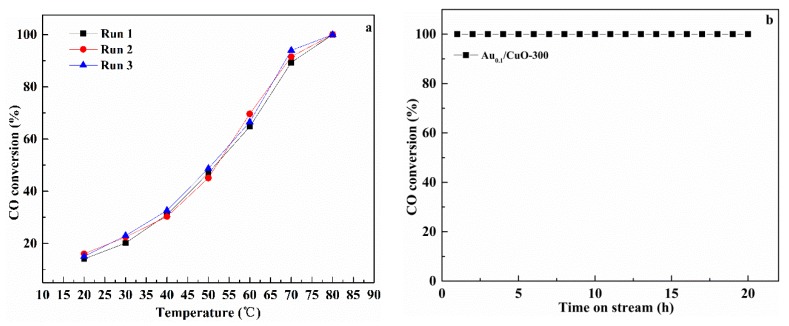
Repeatability of CO oxidation behavior under reaction conditions (**a**) and stability for the CO oxidation at 180 °C (**b**) over Au_1.0_/CuO-300 catalyst.

Figure 9a presents the catalytic reusability of Au_1.0_/CuO-300 for three successive cycles under reaction atmosphere. The corresponding consequences display that the full CO conversion for each run was located at 80 °C, suggesting that Au_1.0_/CuO-300 had well repetitious availability without obvious deactivation in the reaction condition.

As we know, although the high catalytic performance at low temperature is very important for air purification, long lifetime at high temperatures is also needed for automotive exhaust gas catalysts. Therefore, the long-term stability test for Au_1.0_/CuO-300 was carried out at 180 °C for 20 h (Figure 9b). It was shown that CO conversion of Au_1.0_/CuO-300 was maintained at this condition, demonstrating that it had good persistent stability without any deactivation at high reaction temperature.

**Figure 10:**
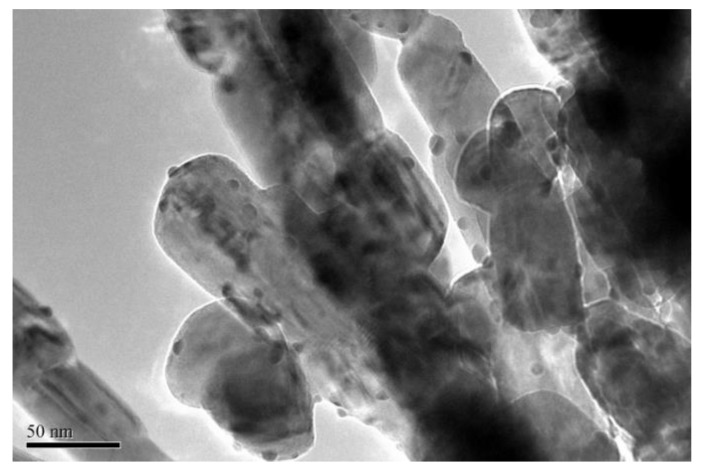
TEM image of Au_1.0_/CuO-300 after the CO oxidation reaction of 20 h at 180 °C.

In order to investigate the reason of stability at high reaction temperature, the gold particle size of Au_1.0_/CuO-300 used for stability test was further examined using TEM analysis (Figure 10). The morphology of Au_1.0_/CuO-300 after the 20-h reaction shows that the gold particles (mean size: 5 nm) were not agglomerate obviously. This is suggesting that its remaining sizes of Au nanoparticles is not only one critical reason for the high stability of catalyst, but also relate with the available synergistic Au-CuO interaction in the used Au_1.0_/CuO-300 catalyst [57]. Moreover, it had been reported previously that the regeneration of active sites in the high reaction temperature could promote the full CO conversion in the low temperature. From the above results, this catalyst has good utilization practically.

Considering economic factors and catalytic performance, urchin-like CuO should be a potential support for gold catalysts. The preparation, active trends, good sinter-resistance and high durability at high reaction temperature for Au_1.0_/CuO-300 in the CO oxidation could provide the new insight for the designing formulations of excellent catalyst. It is generally known that the fabrication parameters could affect the catalytic performance of gold catalyst. Therefore, the catalytic activity of this kind of catalyst could be further optimized deeply. Moreover, many other catalytic reactions could be acted as probe reactions to examine the activity of this catalyst, probably producing new opportunities for the Au/CuO catalyst, application in the future.

#### 3.7.4. Mechanism

**Figure 11:**
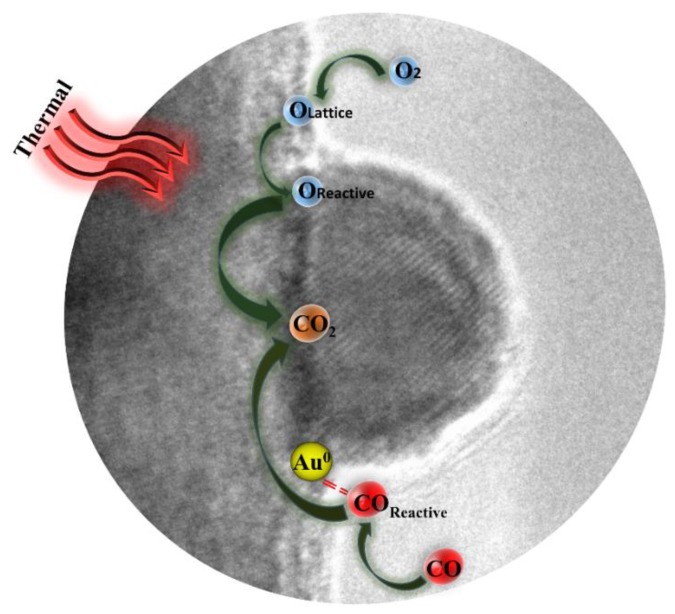
Schematic illustration of possible mechanism for CO oxidation by Au_1.0_/CuO-300 catalyst.

It has been widely proven that several factors (such as gold particle size, nature of support, interface structure, and metal–support interaction) play significant role on the performance of gold catalysts [15,58]. Hence, different mechanisms of CO oxidation over gold catalyst have been suggested in other literatures [59,60]. It was generally accepted that the reaction (CO oxidized to CO_2_) is actually occur on the interface between support and gold particle, which is often considered as active site [61,62]. According to the results above and previous studies, the reasonable catalytic mechanism of Au_1.0_/CuO-300 catalyst for CO oxidation is schematically illustrated in Figure 11. This may shed new explanation on the observation for the high activity of Au_1.0_/CuO-300 catalyst.

Firstly, it is probable that Au sites existed in the Au-CuO interface could preferentially adsorb and activate CO [63]. Accordingly, the interfacial CuO would supply lattice oxygen to be activated and leave oxygen vacancies. Secondly, reactive oxygen species are easily to react with activated CO molecules to generate CO_2_. Thirdly, the gas-phase O_2_ are activated on the interfacial CuO to replenish the oxygen vacancies [64]. Overall, the adsorption and activation of reactant molecules in the gold catalyst are quite important during CO oxidation process [63].

Moreover, the size of gold particle plays the key role in the catalytic activity [65]. The good activity of Au_1.0_/CuO-300 could be interpreted in the light of quantum size effect and structural size effect of gold nanoparticles. The structural size effect is that the small particle size not only contributes to the change of electronic property of surface atoms, but also has more edge and corner atoms on the gold nanoparticle surface, which thus boosts the activation of CO and O_2_ during CO oxidation [66,67]. It is generally believed that small gold particles result in the more interfacial sites, which could accelerate the CO oxidation more efficiently.

## 4. Conclusions

Under the non-hydrothermal condition, the well-defined urchin-like CuO was prepared by optimizing the time of dropping of NaOH. Based on the effect of time of dropping of NaOH on morphology of CuO and related references, its formation mechanism was reasonably proposed. Au_1.0_/CuO-300 catalyst had better catalytic activity and high stability for CO oxidation than other synthesized catalysts. The distributed gold particles with small size on the CuO and synergistic interaction between Au and CuO were the main reason. Combining with the characterization results and activity data of catalysts, the reasonable catalytic mechanism of Au_1.0_/CuO-300 for CO oxidation was proposed. Based on reusability and stability test, the prepared Au_1.0_/CuO-300 catalyst possesses quality utility. The application of the urchin-like CuO—such as photocatalysis, hydrogen production, chemical battery, and solar cells—was anticipated.
